# The Ear and Its Diseases

**Published:** 1888-12

**Authors:** 


THE EAR AND ITS DISEASES, being Practical Contributions to the Study of Otology. By Samuel Sexton, M. D., Aural Surgeon to the New York Eye and Ear Infirmary; Fellow of the American Otological Society; Fellow of the New York Academy of Medicine; Member of the Medical Society of the County of New York and of the Practitioners' Society of New York. Edited by Christopher J. Colles, M. D. Octavo, 473 pages. Numerous illustrations. Extra muslin, $4.00. New York: William Wood & Company.

This work is not a full and comprehensive treatise on diseases of the ear, but one which is devoted more especially to those subjects which appear to have engaged the attention of the author during his long service in the treatment of aural diseases. While we have a number of very valuable text-books from eminent otologists, contributions upon particular topics are always desirable. The practitioner is always anxious to get the views of those whose inclination or position leads them in a particular direction. Such is the vent of the above volume. The reader will find many conditions to which more than usual space and attention are given. There are quite a number of remedies proposed in the treatment of ear diseases which are to be found in no other books, and according to the results of cures published are well worthy of investigation and trial. A number of pages are directed to the excision of the drum-head and ossicles as a curative measure in certain classes of middle ear diseases. The method and instruments are carefully explained and a large number of cases reported. This operation, however, is not as yet accepted by the leading otologists. In a chapter upon the injurious effects of unskillful treatment, the writer is very pronounced in his opposition to the employment of leeches. We do not see how they can be dispensed with in the inflammatory stages of middle ear diseases, and much stress is laid upon their use by the most prominent specialists. Dr. Sexton has had a large experience during a long and valuable service in one of the largest aural institutions in the States. He is a progressive aural surgeon, and his book contains much new and valuable material, not only to the specialist in this branch, but also to the general practitioner.

